# Interactions between polyethylene and polypropylene microplastics and *Spirulina* sp. microalgae in aquatic systems

**DOI:** 10.1016/j.heliyon.2021.e07676

**Published:** 2021-07-30

**Authors:** H. Hadiyanto, Adian Khoironi, Inggar Dianratri, Suherman Suherman, Fuad Muhammad, Seetharaman Vaidyanathan

**Affiliations:** a Center of Biomass and Renewable Energy (CBIORE), Diponegoro University, Semarang, Indonesia; b School of Postgraduate Studies, Diponegoro University, Semarang, Indonesia; c Chemical Engineering Department, Faculty of Engineering, Diponegoro University, Semarang, Indonesia; d Biology Department, Faculty of Science and Mathematics, Diponegoro University, Semarang, Indonesia; e Study Program of Environmental Health, Faculty of Health, Dian Nuswantoro University, Semarang, Indonesia; f Department of Chemical and Biological Engineering, Sheffield University, UK

**Keywords:** Degradation, Microplastics, Polyethylene, Polypropylene, *Spirulina* sp., Phycocyanin

## Abstract

This study aimed to evaluate the effect of microplastics on *Spirulina* sp., the pigment phycocyanin in *Spirulina* sp., and the effect of *Spirulina* sp. on the degradation of PE and PP plastic. The interaction of *Spirulina* sp. with microplstic (PE and PP) was conducted by adding the microplastic (500 mg/500 mL, with a size of 0.5–1 mm^2^) to microalgae culture. The optical density was measured for 30 days to determine the growth of *Spirulina* sp. Harvesting was performed to obtain dry *Spirulina* sp biomass. Phycocyanin was obtained through extraction by mixing 0.1 g dry *Spirulina* sp. biomass with 25 ml of 1% CaCl_2_ in an ultrasonic water bath at 50 kHz, 300 W at 30 °C for 15 min. The results showed that the growth rate of *Spirulina* sp significantly decreased (p < 0.05) with treatment of PE (SP + PE) (0.0228/day) and PP (Sp + PP) (0.0221/day), compared to the control (Sp-Control) (0.0312/day). Scanning electron microscopy and Fourier transform infrared spectroscopy (FTIR) analyses of *Spirulina* sp. biomass with the addition of PE and PP revealed surface damage of *Spirulina* sp. cells and loss of carboxyl groups from proteins in *Spirulina* sp. at wavelengths of 1397–1450 cm^−1^. In addition, *Spirulina* sp. had decreased the intensity of amine and amide groups from proteins at wavelengths of 3280, 1637, and 1537 cm^−1^ in the microplastic treatment. The phycocyanin yield and protein content in *Spirulina* sp. control were 19.69% and 0.147%, respectively, which decreased by 10.7% and 0.121%, respectively, with PE treatment and by 8.7% and 0.108%, respectively, with PP treatment. Moreover, the investigation of PE and PP treated by *Spirulina* sp showed more significant changes of functional group indicated by the formation of hydroxyl (3286 cm^−1^), carbonyl (1700 cm^−1^), ester (1750 cm^−1^) and primary alcohol (1085 cm^−1^). The results of the EDX microplastic analysis showed a decrease in carbon in PE (1.62%) and PP (1.08%). These FTIR and EDX analysis also proved that microplastic has experienced degradation when treated by *Spirulina* sp cell culture.

## 1 Introduction

Polyethylene (PE) and polypropylene (PP) are the most produced and used thermoplastics in the industrial sector. Plastic PE and PP represent up to 85% of the synthetic plastics produced and are mainly used for single-use packaging (Issac and Kandasubramanian, 2021). According to the Indonesian Olefins, Aromatic and Plastics Industry Association, PP production reached 70% in 2019 and PE production almost reached 90% of the total plastic production in Indonesia. PE and PP are polyolefins with a long linear hydrocarbon chain, which makes them difficult to degrade naturally, and it can take more than 100 years to degrade PE and PP plastics (Chamas et al., 2020). The physical and chemical properties of the polymers further affect their degradability. Crystallinity plays a role in polymer strength and stiffness (Sarmah and Rout, 2018, 2019). PE plastic bags have a crystallinity of 60%–80%, and PP used as packaging for mineral water, bottle caps, drinking straws, and others has a crystallinity of 55%–70%. PP has a higher resistance to cracks, acids, organic solvents, and electrolytes and better hydrophobic properties owing to its high molecular weight of 42 g/mol, tensile strength of 31–41 mPa, and hardness of 98%. PE has a lower molecular weight than PP (28 g/mol), tensile strength of 17–33 mPa, and hardness of 60%–69% (Galeski, 2003; Khoironi et al., 2020). Furthermore, PP also has a triple bond (alkyne, C≡C) in its chain structure so that it has greater strength than PE, whereas PE has a double bond (alkene, C=C) (Sutar et al., 2018). These properties confer PE and PP with high stability and strong resistance, which is the primary reason for their use in industries. However, in combination with a long linear hydrocarbon chain, these same properties make the natural decomposition of PE and PP plastics difficult and lengthy.

However, Li et al. (2018) report that PE and PP plastic waste accumulation in water systems eventually degrade into microsized plastics especially due to physical factors. The size of the plastic molecules strongly influences the ability of plastics to release additives (Koelmans et al., 2013). The smaller plastic size affects the increasing number of additives released into the environment. Zhu et al. (2020) and Chae et al. (2019) report that the plastics size affects the ability of microorganisms, especially microalgae, to adsorb additive particles that cause cell membrane damage and growth inhibition. During degradation, plastics release toxic additives that have been added during manufacturing, such as plasticizers, polychlorinated biphenyls, dichlorodiphenyltrichloroethane, and heavy metals such as cadmium, chromium, bromium, copper, and titanium (Campanale et al., 2020). The release of additives or toxic chemical compounds as a result of the degradation process has the potential to be more harmful to the environment (Nava and Leoni, 2021). Capolupo et al. (2020) investigated the effect of chemical additives in plastics on the microalgae *Raphidocelis subcapitata* (freshwater) and *Skeletonema costatum* (marine) and reported that almost all additive particles inhibited algae growth.

Microalgae *Spirulina* sp. are the largest algae-based food products in the United States and Asia. They are generally recognized as safe for consumption and are certified by the Food and Drug Administration (Lucas et al., 2018). *Spirulina* sp contain important organic functional groups such as hydroxyl from polysaccharides and carboxyl from proteins. The protein in *Spirulina* sp. is the blue pigment phycocyanin, a protein composed of a collection of peptides that form polypeptides, with each peptide being composed of amino acids containing a carboxyl group. Besides carboxyl groups, phycocyanin also consists of carbonyl groups, amines, amides, phosphoryl, and sulfonyl (Dmytryk et al., 2014). The pycocyanin is mostly used for its anti-oxidant, anti-cancer, and anti-inflamation properties (Hadiyanto et al., 2016). For food and feed applications, phycocyanin from *Spirulina* sp. extract should contain non-toxic, non-carcinogenic properties and must be free of contaminants, including microplastics (Karthick Raja Namasivayam et al., 2019).

Several studies have shown the effects of microplastics exposure to microalgae based on several effect criteria (Nava and Leoni, 2021). During cultivation, microalgae can produce extracellular polymeric substance (EPS), and the presence of microplastics can stimulate the EPS generation (Song et al., 2020; Chentir et al., 2017). EPS has the potential to form hetero-aggregates with microplastic particles in the biodegradation process (Cunha et al., 2019). The additives released upon plastic degradation also contribute to adhering to the algal cell surface and penetrating the EPS, consequently damaging microalgal cells (Song et al., 2020; Zhu et al., 2020). Restrepo-Florez et al. (2014) reported that the interaction between microorganism colony and plastic result in changes of functional groups and physical properties of plastic. In addition, the increase of carbonyl group intensity and the decrease in molecular weight were observed by Sivan (2011) and Mukherjee et al. (2015), when the microorganism interacted with the polyethylene surface. The interaction also give an impact on PET surface in form of surface erosion as indication of early phase of degradation by *Bacillus subtilis* (Nakkabi et al., 2015). The interaction between *Chlamydomonas reinhardtii* with HDPE and PP microplastics was also investigated by Lagarde et al. (2016), and they found a similar phenomena of surface morphological changes. The reduction of carbon compound in microplastic was also observed by Sarmah and Rout (2018). However, until now, no studies have been reported on the effects of PE and PP microplastics degradation on microalgae *Spirulina* sp. and their effects on phycocyanin in *Spirulina* sp after being contaminated by PE and PP microplastics. Therefore, studies on the interaction between microplastics and *Spirulina* sp., with a focus on phycocyanin will be of value. This study aimed to determine the effect of microplastics PE and PP on *Spirulina* sp., the phycocyanin pigments contained therein, and the role of *Spirulina* sp. in the degradation process of PE and PP plastics.

## 2 Materials and methods

The plastics used in this study were PE obtained from single-use white plastic packaging and PP obtained from single-use mineral water bottles of the brand “AQUA.” Microalgae *Spirulina* sp. culture were obtained from Neoalgae Company (Sukoharjo, Central Java, Indonesia). *Spirulina* sp. cultivation, phycocyanin extraction, and the analysis of the results were conducted at the UPT C-BIORE Laboratory, Diponegoro University, Indonesia. The following treatments were *Spirulina* sp. without microplastic treatment (Sp control), *Spirulina* sp. with PE treatment (Sp + PE), and *Spirulina* sp. with PP treatment (Sp + PP).

### 2.1 Preparation of microplastics

Microplastics were prepared by cutting unused white plastic packaging bags for PE and disposable mineral water bottles for PP. The microplastic was prepared by cutting the plastic bottle to 0.5–1 mm^2^. After the cutting process, the microplastics were washed with ethanol – ethanol aims to clean the contaminants on the plastic – and dried at room temperature for 24 h. Next, 500 mg of PE and 500 mg of PP microplastics were carefully weighed and added into a bioreactor containing 2 L of *Spirulina* sp. culture.

### 2.2 Preparation of *Spirulina* sp.

*Spirulina* sp. was cultivated in three 2-L glass bioreactors, each equipped with an aerator for oxygen supply and illuminated by conventional LED lights (3000 lux). The nutrition comprised a mixture of 15 ppm *Triple Super Phosphate (TSP*), 70 ppm urea, and 1 g/L NaHCO_3_ and was administered every 5 days. For 30 days, pH was maintained at 7–8 and temperature at 24°C–26 °C. The optical density (OD) was measured using a spectrophotometer (OPTIMA SP-300) to determine cell concentrations in *Spirulina* sp. Chlorophyll-α levels were determined at a wavelength of 680 nm using ultrapure water as a blank solution. Increasing chlorophyll-α levels indicated growth of Spirulina sp. On the first day, OD was measured and obtained a value of 0.42 for the three variables.

### 2.3 Harvesting of *Spirulina* sp.

Stainless steel wire-mesh screens of 40 μm were used during the harvesting process. This filters were used to filter the microplastics out of the mixture, where the biomass and filtrate were separated (Khademi, 2014). Finally, the wet biomass of *Spirulina* sp. was dried at 30°C–35 °C for 24 h to get the dry biomass of *Spirulina* sp. After obtaining the dry biomass of Spirulina sp., the extraction process was carried out.

### 2.4 Extraction of phycocyanin

The extraction of phycocyanin from *Spirulina* sp. was performed using a Krisbow ultrasonic cleaner (tool item #10039597) and a method conducted by İlter et al. (2018) and Prabuthas et al. (2011) was used. First, a glass beaker was filled with a mixture of 0.1 g of dry *Spirulina* sp. biomass and 25 ml 1% CaCl_2_ solvent and it was then covered with aluminum foil. The three glass beakers was placed in an ultrasonic water bath filled with aquadest, and the ultrasonic treatment operated for 15 min, with heating power 300 W, ultrasound frequency 50 kHz, and a bath temperature at 30 °C. As the phycocyanin dissolved into the solvent, it accumulated into the supernatant, which was then separated from the *Spirulina* sp. residue in a centrifuge at speed 4,000 rpm for 15 min. In this extraction method, the ultrasonic waves use liquid as a propagation medium, which increases the intensity of energy transfer, thereby maximizing the yield of the extraction process compared with conventional methods (Shirsath et al., 2012).

### 2.5 Phycocyanin analysis

Analysis of proteins of phycocyanin from *Spirulina* sp. was performed at the Center for Industrial Pollution Prevention Technology, Semarang, Indonesia (SNI 01-2891-1992 item 7.1). The phycocyanin yield, purity, and concentration were determined using a spectrophotometer (Spectroquant Prove 100). Phycocyanin was noted at a peak of 620 nm. Its purity was calculated based on the absorbance ratio of 620/280 nm (Pagels et al., 2019). The concentration of phycocyanin was measured at absorbances of 280, 620, and 652 nm. Finally, the concentration was measured in accordance with the procedure described by Brandt et al. (1989):

$$PC=\frac{OD_{620}-\;0.474OD_{652}}{5.34}$$

where *PC* is the concentration of phycocyanin (mg/mL) and *OD*_*620*_ is the amount of phycocyanin present in the *Spirulina* sp extract. The alophycocyanin content in the extract is given by *OD*_*652*_. Using this formula, we obtained the formula for phycocyanin yield. Extraction yield was defined in accordance with the procedure described by Silveira et al. (2007):

$$\text{Yield}=\frac{PC\times V}{DB}$$

where *yield* is the result of phycocyanin extraction expressed in milligrams of phycocyanin per gram of dry biomass (%), *V* is the volume of the solvent (mL), and *DB* is the dry biomass (g).

### 2.6 Fourier transform infrared spectroscopy (FTIR) and scanning electron microscopy (SEM) analyses

FTIR analysis was performed using dried *Spirulina* sp. biomass. The purpose of FTIR analysis was to determine the effect of microplastics on the content of organic groups in *Spirulina* sp., especially the carboxyl, amine, and amide groups, which are indicators of the formation of phycocyanin protein compounds. FTIR analysis was performed to evaluate the changes in polymer functional groups and SEM analysis as well as to observe changes in the surface morphology of *Spirulina* sp. and microplastics. Energy-dispersive x-ray (EDX) analysis was conducted to assess the changes in the composition of PE and PP organic elements due to the degradation process of *Spirulina* sp. (Dianratri et al., 2020).

### 2.7 Statistical analysis

The experiment was applied in triplicates and the results were as means ± standard error of the mean. Prior to AOVA analysis, the t-test for two-sample with unequal variances was conducted to evaluate the differences of their means. Then, analysis of variance was performed by one-way ANOVA, following Tukey's HSD (honest significant difference) Post Hoc multiple comparison analysis, using GradPrism 9. A value of p < 0.05 was used to determine a significant difference.

## 3 Results and discussion

### 3.1 Influence of microplastics on Spirulina sp. growth

**Figure 1:**
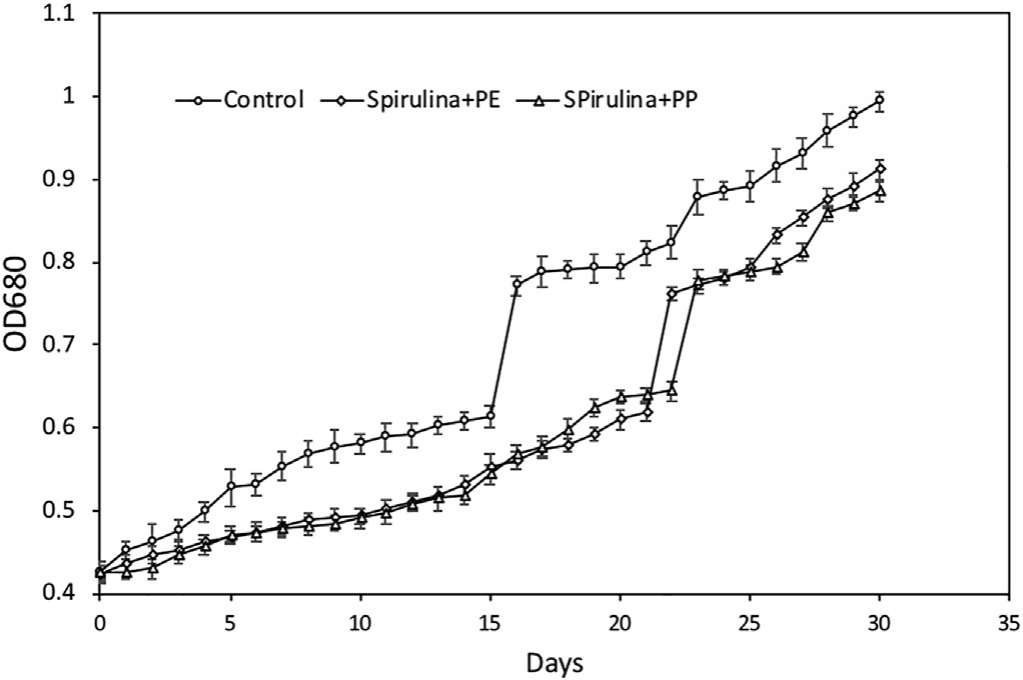
Microalgal growth of *Spirulina* for all three treatments with and without (control) microplastic. The error bars were standard error (n = 3).

The results show that OD_max_ of *Spirulina* sp. without microplastic treatment in (Figure 1) was 0.994 ± 0.015 higher than that of *Spirulina* sp. treated with PE and PP microplastics, i.e., 0.912 ± 0.021 and 0.886 ± 0.009 (Dianratri et al., 2020). The results in Figure 1 also confirm that the growth rate of *Spirulina* sp. without microplastic treatment was higher than that of *Spirulina* sp. treated with PE and PP microplastics, i.e., 0.0312, 0.0221, and 0.0228 day^−1^, respectively.

The decreased microalgal growth due to the presence of microplastic is statistically significant, (p < 0.05) as shown in (Table 1). From the t-test with unequal variance statistical analysis, it is shown that there was significant difference of population means between Sp-control and Sp + PE (t-critical two tail (2.31)<t_stat_ (4.96)) and also Sp-control and Sp + PP (t-critical two tail (2.31)<t_stat_ (6.41)). However, there was no significant difference of growth rate of microalgae treated with Polyethylene (PE) and Polypropylene (PP) ((t-critical two tail (2.31)>t_stat_ (1.35)).

**Table 1:** The growth rate and OD_max_ of *Spirulina* sp. over a period of 30 days. The letters indicate significance of different treatments between different type of microplastic applied (P < 0.05). Values represent mean ± standard error (n = 3).

System	OD_max_	Growth rate (day^−1^)
Sp control	0.994 ± 0.015	0.0312 ± 0.0076^a^
Sp + PE	0.912 ± 0.021	0.0221 ± 0.0081^b^
Sp + PP	0.886 ± 0.009	0.0228 ± 0.0074^b^

The presence of microplastics can interfere with the intensity of light that enters the *Spirulina* sp. culture for photosynthesis (Reichelt and Gorokhova, 2020). Inhibition of photosynthesis results in decreased oxygen production, leading to reduced microalgae density in the culture. This finding was supported by Li et al. (2020), who reported that over a period of 6 days, the presence of microplastics in microalgal cultures caused their density to decrease, the chlorophyll level to reduce, and their surfaces to be damaged, resulting in a reduction in growth rates of 45.8%. The damaged surfaces were reported by Zhu et al. (2020), who stated that additive compounds released by microplastics become highly toxic on their interaction with *Spirulina* sp. These toxic additives then damage the cell membranes of *Spirulina* sp. and reduce their growth rate. Barreto et al. (2019) and Zhu et al. (2020), reported that particles of microplastics such as ions in heavy metals are factors that cause toxicity. The ions could inhibit the photosynthesis process and induce oxidative stress response, resulting in the growth inhibition of algae. Similarly, PE and PP microplastics, which have additive particles such as copper, titanium, bromium cause the growth of *Spirulina* sp to decrease.

In contrast, the growth rate of *Spirulina* sp. in the presence of microplastics continued to increase, showing the adaptability of *Spirulina* sp. as it utilized microplastics as a carbon source for growth (Sarmah and Rout, 2019; Khoironi et al., 2019). In the interaction of thermoplastic and algal, algae cells can capture and store carbon dioxide in biomass form permanently. Consequently, carbon dioxide will not be emitted back into the environment (Hadiyanto et al., 2012). Tian et al. (2014) investigated the growth phases of microorganisms and demonstrated their ability to survive in stressful conditions caused by the presence of microplastics. On comparing the growth rate of *Spirulina* sp. affected by microplastics, there was no differences of growth rate of *Spirulina* sp. affected by PE and by PP microplastics (Table 1). However, the maximum biomass achieved by *Spirulina* cultivated in PE microplastic (OD = 0.916) is higher than those cultivated in PP (OD = 0.886). This may be because PE is more easily decomposed than PP, so that PE releases carbon faster and is used by *Spirulina* sp. for photosynthesis (Lagarde et al., 2016).

### 3.2 SEM analysis to evaluate changes in the surface morphology of Spirulina sp. and microplastic

**Figure 2:**
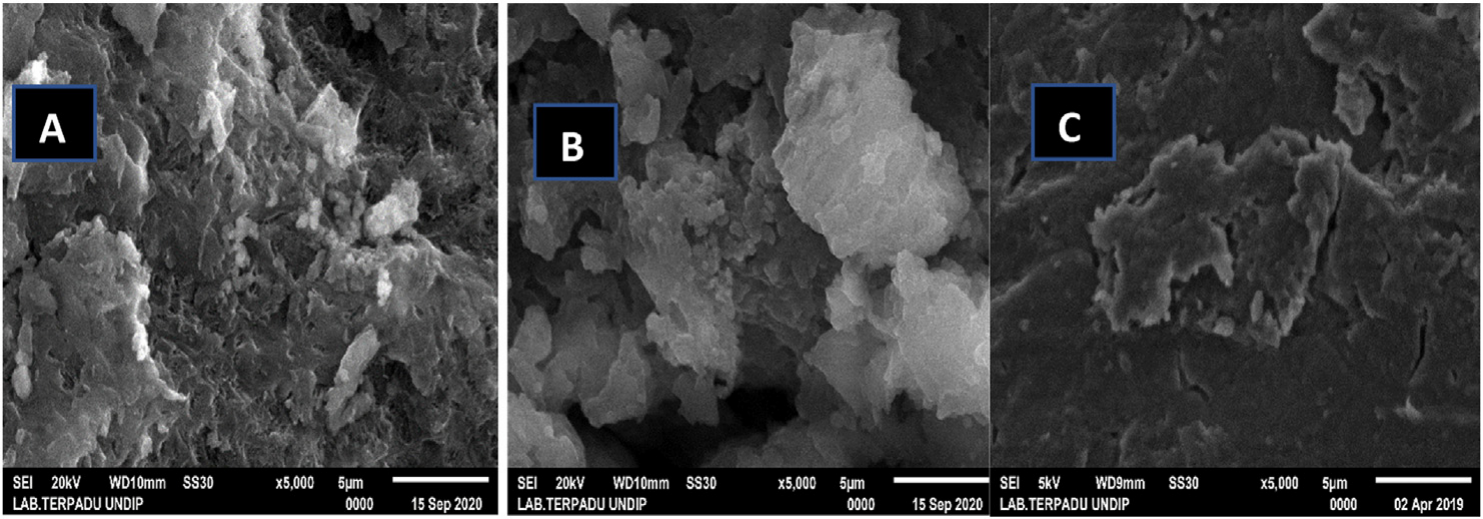
SEM analysis results of the surface morphology of *Spirulina* sp. over 30 days. *Spirulina* sp. control (A), *Spirulina* sp. + PE microplastic (B), *Spirulina* sp. + PP microplastic (C).

The damage caused to *Spirulina* sp. cell walls after interaction with microplastics was visualized using SEM analysis over a period of 30 days and shown in Figure 2. The results of SEM analysis showed morphological changes in *Spirulina* sp. with and without microplastic treatment in Figure 2. In *Spirulina* sp without microplastic treatment, the resulting EPS production was under normal conditions because there were no threatening foreign bodies in the culture (Vimal Kumar et al., 2017). Microplastics are used by microalgae as a carbon source and their presence results in increased production of EPS, indicating that microalgae do experience stress (Sarmah and Rout, 2019). In Figure 2A, *Spirulina* sp-control have a plain appearance and are surrounded by EPS. Damage to *Spirulina* sp. cells due to the presence of microplastic particles can be seen on the uneven cell surface compared to *Spirulina* sp. control. In *Spirulina* sp. with microplastic treatment, the EPS production increased as shown in Figures 2B and C, and the EPS production in *Spirulina* sp. with PE microplastic treatment appeared to be higher than *Spirulina* sp with PP treatment. These results are in accordance with the research of Lagarde et al. (2016), who reported that microalgae genes were involved in EPS production, and that microalgae EPS production with HDPE treatment was more dominant than PP treatment. Song et al. (2020) reported that the addition of microalgae EPS production indicates a form of protection for microalgae from contaminants including microplastic additive particles.

In contrast, SEM results of PP microplastics used in this study better resembles the surface shape of *Spirulina* sp. control, but has a flake-like shape. An uneven surface indicated surface erosion, and the formation of holes and disruption confirmed the more significant fragility of the *Spirulina* sp with PP treatment. This shows that additives released from PP microplastics enter and damage the cell surface. Damage to the surface of microalgae cells is caused by the presence of foreign bodies as contaminants in culture. Several studies have reported that microplastic particles inflict direct physical damage on algal cells, thereby inhibiting their growth. Song et al. (2020) reported in their results that there was severe damage to the cell surface structure of *Phaeodactylum tricornutum* and that microplastic particles were found to aggregate outside the cell. Zhu et al. (2020) compared the surface damage of *S. costatum* algae cells by PVC microplastics and nano-Cu additive particles. Microalgae were able to absorb both microplastics and nano-Cu onto the cell surface, enveloping the algal cell surface and causing mechanical damage or oxidative damage (Bellingeri et al., 2019). They concluded that microplastics and Cu nanoparticles had a toxic effect on *S. costatum*, and the toxicity of nano-Cu on algae was higher than that of microplastics. Both nano-Cu and microplastics were adsorbed by *S. costatum*, resulting in the cells membrane damaged and growth inhibition (Song et al., 2020; Zhu et al., 2020).

Furthermore, EPS was not abundant in microalgal cells treated with PP, leaving *Spirulina* sp. less protected against additives. The difference in EPS abundance is because of the better degradability of PE microplastics (Lagarde et al., 2016). PE can release more carbon, which microalgae use to form additional EPS. PP tends to be hydrophobic and stronger but has lower degradability than PE. The carbon used by microalgae on PP tends to result in less EPS production and facilitates the entry of additive particles from microplastics into cells (Song et al., 2020; Zhu et al., 2020). The differences in EPS production are in accordance with the results reported by Lagarde et al. (2016), who studied interactions between *Chlamydomas reinhardtii* with high-density PE (HDPE) and PP microplastics. Their results showed higher EPS production following interaction with HDPE than that after interaction with PP. Furthermore, *Spirulina* sp. has a fragile cell wall and may appear with many cavities because of damage by microplastics. This is evident in the number of microplastic particles and additives that managed to enter and adhere to microalgal cells. Oxidized microplastics in *Spirulina* sp. culture may release its additive particles in the form of ions which were much smaller than the microplastics themselves. Although it was not in our investigation, this phenomena was suggested by Song et al. (2020), who described the additive particles attached to the surface of microalgae cell covered by EPS as a form of protection. Wang et al. (2017) reported that EPS in small amounts is less effective in protecting the cell membrane from contamination, resulting in easier entry for microplastic additives and eventually damaging the cell membrane structure.

**Figure 3:**
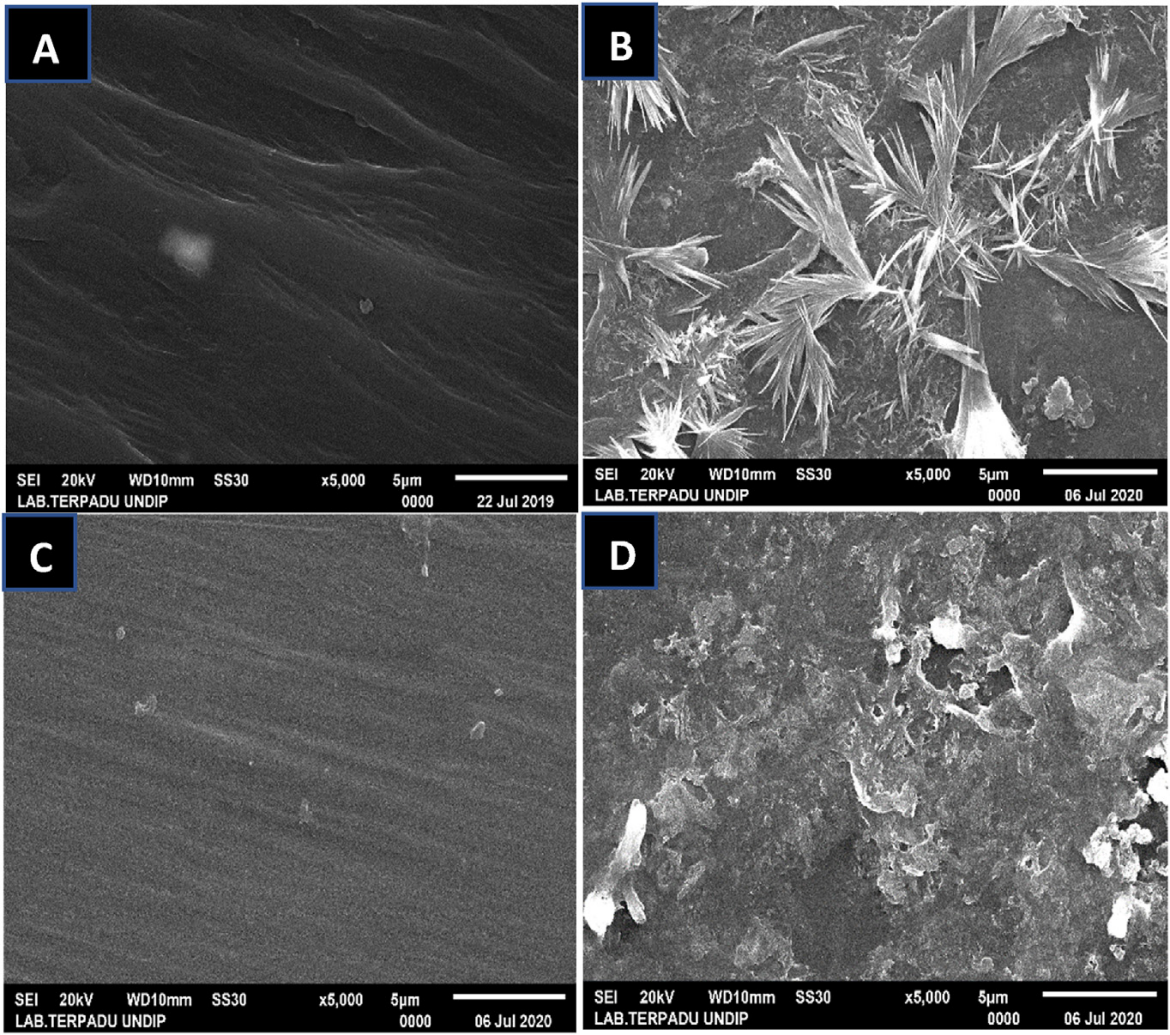
SEM analysis results of the surface morphology of microplastics. PE without treatment (A), PE treated with *Spirulina* sp. (B), PP without treatment (C), and PP treated with *Spirulina* sp. (D).

The SEM analysis results of the surface morphology of microplastics are shown in Figure 3. As shown in Figure 3A, B, C, and D, a smoother surface was observed on PE than on PP before treatment. The surface of PP was rougher and less even. This is due to the hydrophobic nature and higher molecular weight of PP and hence, the surface roughness of PP is higher than that of PE. Hydrophobicity can be measured by the contact angle of water on the solid surface. A surface is called hydrophobic if the contact angle of water on it is greater than 90°. Conversely, if the contact angle of water on it is less than 90°, the surface is hydrophilic (Sakti et al., 2017). PE's contact angle of water is 86–94° and for PP, this is 95°. This indicates that PP tends to be more hydrophobic (Myshkin and Kovalev, 2018). Furthermore, Sakti et al. (2017) have reported that hydrophobicity also plays a role in the immobilization of biomolecules. The higher the molecular weight (MW), the better the level of immobilization of the molecules, and the added hydrophobic properties of the plastic (MW PE 28 g/mol, MW PP 42.07 g/mol) (Sutar et al., 2018).

After treatment with *Spirulina* sp., the SEM results of PE revealed many cracks in the form of crystals filled with a white substance. This white substance was EPS slime produced by *Spirulina* sp., which plays an important role in the biodegradation process by adhering to and damaging the surface of PE microplastics (Lagarde et al., 2016). *Spirulina* sp. produces EPS when it digests any nutrient in culture, including microplastics. When *Spirulina* sp. produces EPS, it accumulates and forms a biofilm on the surface of any solid object in contact with *Spirulina* sp., including microplastics in culture. The biofilm will be utilized by bacteria/fungi/microorganisms that play a role in the degradation process of the microplastic surface. This can be seen in the SEM results, where the surface of the plastic looks like a crack. This crack is an indicator that the degradation process occurs due to the presence of EPS, which forms a biofilm on microplastics surface (Restrepo-Flórez et al., 2014). Restrepo-Flórez et al. (2014) add that PE is composed of crystalline and amorphous regions. Microorganisms are reported to prefer amorphous regions. Corrosion of PE surfaces by cyanobacteria appears to be scattered and non-uniform, indicating that amorphous regions of the polymer were more susceptible to degradation by cyanobacteria. When the more accessible amorphous regions are depleted, microorganisms continue on smaller crystals, resulting in an increased proportion of larger crystals (Arutchelvi et al., 2008). This supports the SEM results in that PE appears to be dominated by a crystalline form and provides evidence of PE biodegradation into its monomeric components. Damage to the PE surface indicates the fragility of PE, following the interaction with *Spirulina* sp. However, erosion and blistering were uneven on the PP surface owing to stronger hydrophobic properties, rendering its surface more resilient. The morphological shape of PP is similar due to damage caused by mechanical friction, which may be caused by several factors, including the use of an aerator in the stirring process. The SEM results of PE and PP indicate that microorganisms and EPS play an important role in the biodegradation process of polymers.

In addition to SEM, which is used to visualize the polymer biodegradation process, EDX analysis was performed to identify the inorganic elements distributed in the polymer following interaction with *Spirulina* sp. The results of the EDX analysis are shown in (Table 2).

**Table 2:** Results of the EDX analysis of the elemental composition in PE and PP before and after treatment with *Spirulina* sp.

Compound	PE		PP	
	Before (%)	After (%)	Before (%)	After (%)
Carbon	97.72 ± 0.07	96.1 ± 0.02	99.64 ± 0.01	98.56 ± 0.01
Calcium oxide	1.01 ± 0.01	-	-	0.18 ± 0.025
Titanium dioxide	0.85 ± 0.01	1.62 ± 0.02	-	-
Copper (II) oxide	0.41 ± 0.02	0.41 ± 0.08	0.36 ± 0.025	0.3 ± 0.012
Sodium oxide	-	1.17 ± 0.075	-	0.37 ± 0.014
Zinc oxide	-	0.24 ± 0.081	-	-
Zirconium dioxide	-	0.46 ± 0.082	-	0.59 ± 0.011

Both PE and PP microplastics experienced only a slight decrease in carbon: 1.62% in PE and 1.08% in PP (p > 0.05). EDX results which showed a decrease in carbon and compound changes in microplastics after treatment did not show a significant difference. The percentage of carbon reduction in PP was smaller than that in PE due to the hydrophobic properties of PP. One of the causes for this is the characteristics of each microplastic in degradation. According to Singh and Sharma (2008), polymer degradation is preceded by an oxidation and hydrolysis process from water to enable it to damage or introduce gaps within hydrophobic polymers. PP is more difficult to oxidize and, accordingly, its hydrolysis is more difficult. Owing to the hydrophobic nature of PP, a longer time is needed for its hydrolysis and for the formation of gaps or holes. This is in contrast with PE, whose surface is more easily damaged by hydrolysis (Gewert et al., 2015). The PE surface is more easily damaged and releases its carbon content more easily, whereas the surface of PP is more difficult to damage, and it releases less carbon than PE. After the gaps are formed, biodegradation occurs and microorganisms start utilizing the carbon from the microplastics as nutrients and produce EPS. This is supported by Sheng et al. (2010), who stated that EPS on biofilms formed on the surface of plastics has the ability to absorb metals and organic compounds. EPS will then help in attacking and destroying the hydrophobic properties of the plastic and increase its hydrophilic properties. In addition, EPS also helps break down more complex polymer chain bonds to produce shorter structure chains that can undergo degradation (Gu, 2003).

The EDX analysis results proved that *Spirulina* sp. degraded microplastics following interaction for 30 days. This finding corroborates those of Sarmah and Rout (2018), who found that after interacting with microalgae over 42 days of cultivation, PE microplastics released 4% carbon. Research by Khoironi et al. (2019) showed that following interaction with *Spirulina* sp. over 112 days of cultivation, PET microplastics released 48.61% carbon, and PP microplastics released 36.7% carbon. Sarmah and Rout (2019) reported that the interaction time affects the amount of carbon released and then utilized by Cyanobacteria to absorb carbon in their biomass. Furthermore, some new inorganic elements appearing or missing are identified in plastic after the treatment of *Spirulina* sp. This is because plastics consisting of chemical additives that are added during the manufacturing process (including Ca, Na, K, Zr, and Zn) have the ability to release and distribute these chemical compounds into culture as harmful contaminants. Plastics are also able to absorb other compounds from the culture, such as nutrients added to the culture of *Spirulina* sp. (Khoironi et al., 2019; Teuten et al., 2009). This inorganic element can be derived from nutrients added to *Spirulina* sp. media and from the release of additive compounds from plastic added during the manufacturing process plastic itself. Rummel et al. (2017) added that the distribution of these chemical pollutants can be through biofilms that are formed on the plastic surface when the aggregation process is in progress.

### 3.3 FTIR analysis to evaluate the changes in the organic functional groups of Spirulina sp. and microplastics

**Figure 4:**
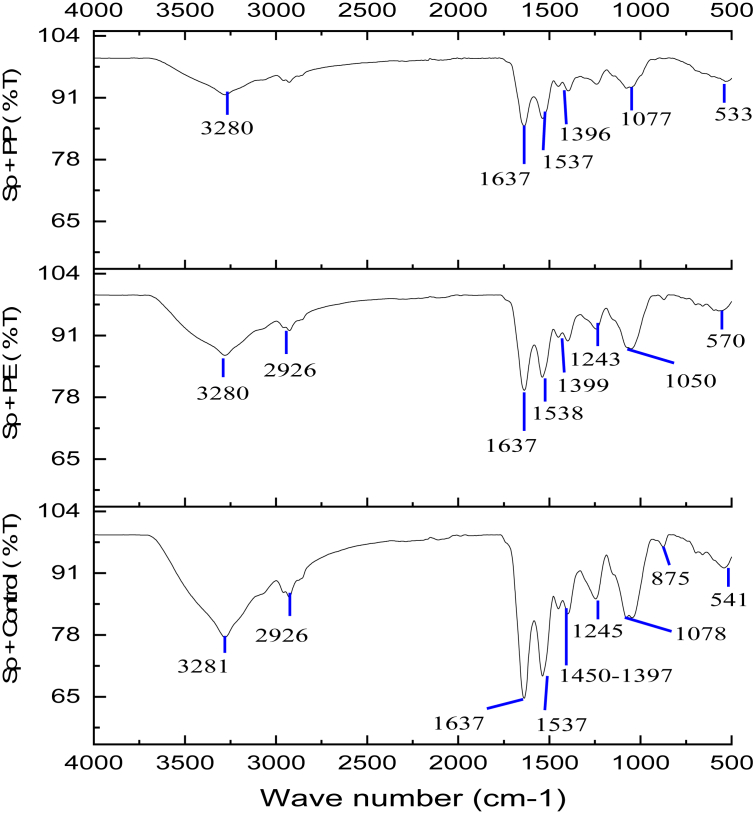
FTIR analysis of *Spirulina* sp. control, *Spirulina* sp. + PE treatment, and *Spirulina* sp. + PP treatment (Dianratri et al., 2020).

The FTIR results in Figure 4 show an increase in the percentage of transmittance (%T), indicating that the intensity of the components at all peaks decreased with PE treatment, and the intensity of some peaks were not detected by PP treatment. At a wavelength of 3281 cm^−1^ a decrease in the intensity of the protein amine component (NH_3_) is indicated, after the interaction with PE and PP microplastics. Likewise, at wavelength 1637 cm^−1^ there was a decrease in the intensity of the secondary amide protein component (C–N) and a carbonyl group (C=O). Dmytryk et al. (2014) similarly reported that the stretching of the primary amide protein components indicates the presence of carbonyl groups at 1650 cm^−1^. Both amine and amide protein intensities in *Spirulina* sp. treated with PE were higher than those treated with PP. The carboxylate group (COO-) of protein at 1450 to 1397 cm^−1^ in the *Spirulina* sp. control group (83.51%) disappeared following PE and PP treatment. Besides the decreasing protein intensity, the intensity of the hydroxyl group (O–H) of polysaccharides also decreased and even disappeared. A study by Dianratri et al. (2020) reported changes in peaks at 1078 and 1245 cm^−1^, indicating the presence of a hydroxyl group in polysaccharides, decreased intensity with PE treatment, and decreased and eventually disappeared intensity following PP treatment. Similarly, wavelength 875 cm^−1^ indicated the presence of polysaccharides and proteins in the phosphoryl (P–O) and sulfonate (SO_3_) groups in *Spirulina* sp. Both disappeared following PE and PP treatment. *Spirulina* sp. treated with PE lost one polysaccharide peak at 875 cm^−1^, and PP treatment led to the loss of two polysaccharide peaks at 1245 and 875 cm^−1^. The FTIR results showed that the damage to *Spirulina* sp. cells caused by microplastics could reduce the quality of *Spirulina* sp. biomass, reduce the productivity of protein and polysaccharides, and even lead to their disappearance (Dianratri et al., 2020).

The decreased quality of biomass because of cell damage stems from the biosorption properties of *Spirulina* sp. (Dmytryk et al., 2014). Biosorption is the ability to absorb carbon contaminants and heavy metal additives, such as cadmium, chromium, cobalt, titanium, and copper, released during the biodegradation process. Heavy metals are often removed with *Spirulina* sp. because it biosorbs Cr^3+^, Cd^2+^, Cu^2+^, and Ti^2+^ ions (Biologi et al., 2014). Its capacity to bind and absorb heavy metals is high because of its functional groups found in the cell wall and cytoplasm. Such functional groups are carboxyl, hydroxyl, amines, sulfonates, and phosphoryl. The carboxyl and hydroxyl protein groups from polysaccharides have negative ions (anions), whereas the heavy metals in microplastics have positive ions (cations), allowing them to bind. This bonding causes damage to *Spirulina* sp. cells and decreases the biomass quality, especially the protein and polysaccharide content. This is in line with Sadiq et al. (2011) and Clément et al. (2013), who studied the interaction between the microalgae *Chlorella* sp. and *Scenedesmus* sp. and TiO_2_, an additive in microplastics. They noted peak changes in the carbonyl, amine, hydroxyl, and carboxyl groups after TiO_2_ metal interaction. Dmytryk et al. (2014) studied the interactions between *Spirulina* sp. and heavy metal copper, another additive in microplastic. They noted a shift in the peaks of the amine, amide, and carboxyl groups and a peak shift at wavelength 1300 to 1000 cm^−1^, showing the presence of –OH, meaning that the hydroxyl group interacted with metal ions because hydroxyl is one of the main binding sites on the surface of the biomass. They did not detect the phosphoryl or sulfone groups introduced by Cu(II) at peak 830 to 862 cm^−1^.

The FTIR results proved that *Spirulina* sp. exposed to microplastics experiences decreased quality of *Spirulina* sp. compared to the *Spirulina* sp. control. However, *Spirulina* sp. contaminated with PE microplastic had a higher biomass quality than contaminated PP microplastic. In PE treatment, the intensity of the content of organic functional groups such as protein and polysaccharides was still higher than *Spirulina* sp after PP treatment. The results of *Spirulina* sp with PP treatment showed that the intensity of the polysaccharide and protein showed a smaller quantity amount and even was not detected. This proves that damage to *Spirulina* sp. cells by PP microplastic was greater than that by PE microplastic. The SEM analysis supported these results, as it showed damage to the cell surface inflicted by microplastics.

**Figure 5:**
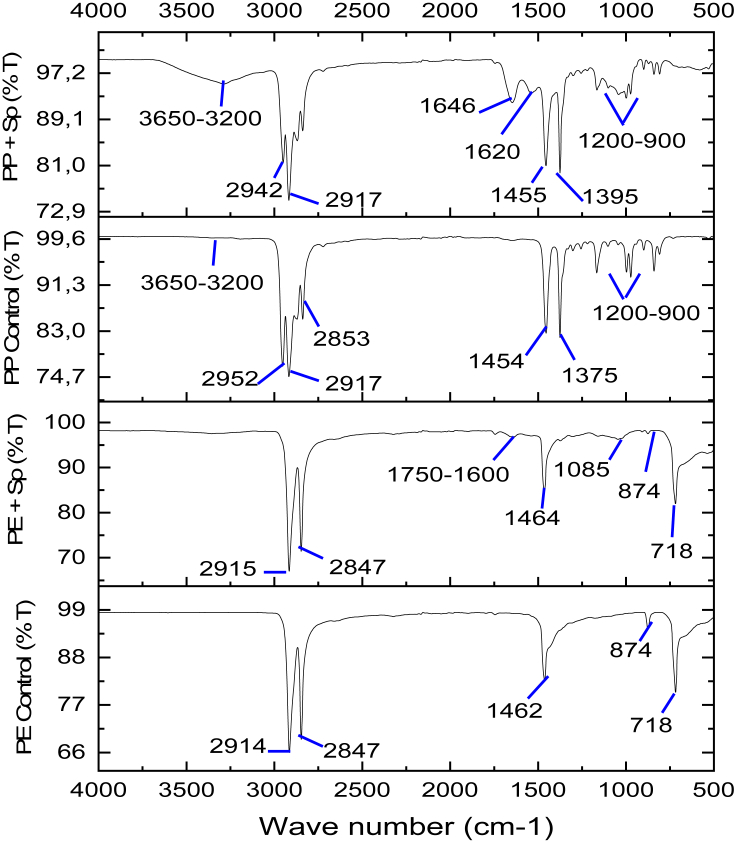
FTIR results of PE control, PE treated with *Spirulina* sp., PP control, and PP treated with *Spirulina* sp.

FTIR results of PE microplastic after *Spirulina* sp. treatment in Figure 5 showed increased %T due to the stretching of alkyl groups (C–H and CH_2_) at wavelength 2950 to 2800 and wavelength 718 cm^−1^. Furthermore, wavelength at 874.94 cm^−1^ shows the loss of alkyl groups. According to Gautam et al. (2007), the presence of peaks in the graphs show alkyl groups (C–H and CH_2_), and wavelength 2950 to 2800 cm^−1^ indicates that the plastic polymer is composed of repeated CH_2_ polymers that form polymer chains. Meanwhile, the covalently bonded C–H group indicates that the plastic has high stability and nonpolar properties, making it difficult to degrade. Therefore, the decrease and even disappearance of intensity in the alkyl groups indicates degradation, as with alkenes (C=C) at wavelength 1462.92 cm^−1^, which experienced an increase in %T, causing the intensity of the component to decrease and almost disappear. New appearances are the carbonyl group (C=O), carboxylic acid (-COOH), and ester at wavelength 1750 to 1600 cm^−1^. The formation of the carbonyl group indicates bond cleavage (C–C), forming a polymer with a low molecular weight to allow it to degrade. This is consistent with Sivan (2011), in that oxidation degradation can reduce the molecular weight of plastic polymers and produce oxygenated groups, such as carbonyl. At wavelength 1085 cm^−1^, a primary alcohol group (C–O) formed. Wavelength of 874.94 cm^−1^ showed the loss of alkyl groups (C–H), and wavelength of 718 cm^−1^ showed an increase in %T due to stretching alkyl groups after PE interaction with *Spirulina* sp. This is because the alkyl groups have non-polar properties and are difficult to degrade. The degradation process is outlined by the decreasing and eventually disappearing intensity in the alkyl groups. The change in the functional groups in PE microplastic shows that it underwent degradation as evidenced by the increase in %T, loss of alkyl groups, increase in %T of alkene (C=C) groups, and emergence of new groups. This result is in line with the recent study of Hervé et al. (2020), who report that the degradation of PE microplastic forms carbonyls as the main product and carboxylic acids and esters as byproducts.

FTIR results of PP microplastic after *Spirulina* sp. treatment in Figure 5 show a sharp peak at wavelength 1646.47 cm^−1^, indicating the presence of the carbonyl group (C=O) that formed after the interaction. The emergence of carbonyl groups may trigger the cutting of PP polymer rings (chain scission) and cross-linking, while reducing the hydrophobic properties of PP (Longo et al., 2011). After that, free radicals from alkyl in the presence of O_2_ from photosynthesis by *Spirulina* sp. caused a reaction with O_2_ to form hydroperoxide compounds and new groups, such as hydroxyl groups, present at wavelength 3286 cm^−1^. Then, at wavelengths 2950 to 2900 and 1459 to 1300 cm^−1^, an increased %T of the alkane group (C–H) is shown, indicating that the intensity of the components in the alkane group is decreasing. Furthermore, at wavelength 1680 to 1620 cm^−1^, another new group is formed, namely an alkene (C=C). The appearance of an alkene group (C=C) indicates that, in PP, which has a triple bond, the degradation process is taking place. Finally, at wavelength 1200 to 900 cm^−1^, a new vinyl group is formed. These results indicated that PP microplastics had undergone oxidative degradation as it is evidenced by a decrease in the intensity of several groups and the emergence of carbonyl, hydroxyl, and vinyl groups. The results of this study are supported by Moldovan et al. (2012), who stated that the appearance of peaks at 1850 to 1630 cm^−1^ (carbonyl), which may trigger chain scission and crosslinking, 3650 to 3200 cm^−1^ (hydroxyl), and 1200 to 908 cm^−1^ (vinyl), is an important product of PP degradation. Gewert et al. (2015) showed the formation of new groups, especially carbonyl, hydroxyl, and vinyl, as the main effect of degradation in PP microplastics. The success of *Spirulina* sp. in degrading PE and PP microplastics is proven by the SEM analysis that showed damage to both PE and PP microplastics.

### 3.4 Effect of microplastics on the purity, yield, and protein values of phycocyanin in Spirulina sp.

**Figure 6:**
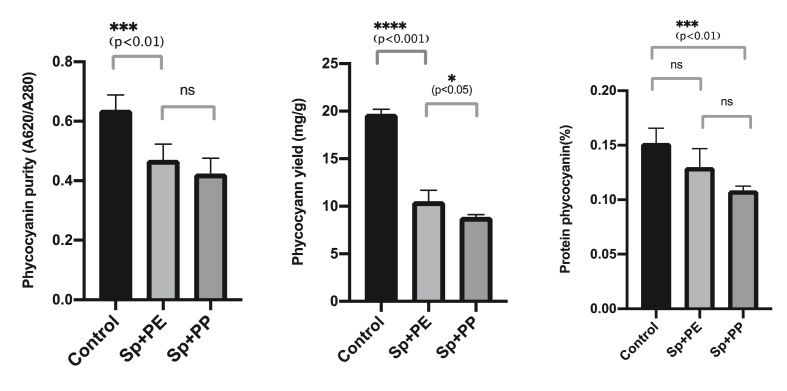
Purity, yield, and protein contents of phycocyanin from *Spirulina* sp. control (a), *Spirulina* sp. + PE treatment (b), and *Spirulina* sp. + PP treatment (c). Significant differences between treatments for each microplastics concentration were noted with asterisks. The error bars were standard error (n = 3).

The purity, yield, and protein content of phycocyanin extracted from *Spirulina* sp. are shown in Figure 6. Figure 6a shows that the microplastic PE and PP also reduce the purity of phycocyanin (p < 0.05), however, the type of microplastic materials did not give significant effect to the purity (p > 0.05). Furthermore, the amount of phycocyanin was also influenced by the amount of yield after the extraction process (p < 0.001). The presence of microplastics interferes with the distribution of light that enters the culture for photosynthesis, causing the growth of *Spirulina* sp to be inhibited (Song et al., 2020). The microalgae growth was inhibited by microplastic and therefore, the biomass and the extracted phycocyanin were also decreased (Figure 6b). Very small particles may be more likely to inhibit the growth of microalgae through adsorption on the surface of the algal cell; for instance, inducing shading, blocking algal pores or gas exchanges, and embedding in microalgae cells (Fu et al., 2019).

**Figure 7:**
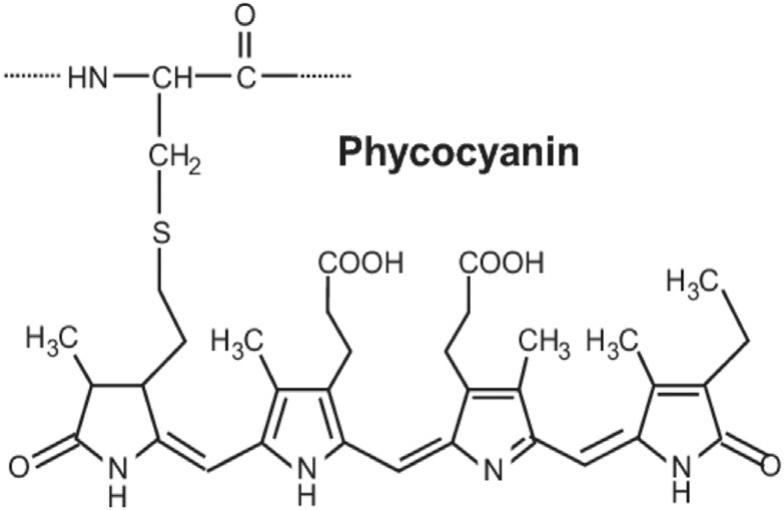
Structure of phycocyanin Zheng et al. (2013).

The structure of phycocyanin comprises several important groups, namely carboxyl groups (COO-), sulfones (SO3), carbonyl (C=O), amines (NH_3_), and amides (CN, NH), as shown in (Figure 7).

The decrease in phycocyanin in *Spirulina* sp. is associated with the FTIR results of *Spirulina* sp. Each of the phycocyanin's peptides is composed of amino acids containing a carboxyl group. The results of the FTIR analysis of *Spirulina* sp. biomass are shown in (Figure 4) (Dianratri et al., 2020). Wavelength 1450 to 1397 cm^−1^ indicated the loss of carboxyl groups, and a sulfone group was lost at 875 cm^−1^. These results are in accordance with Dmytryk et al. (2014), who studied interactions between *Spirulina* sp. and the heavy metal copper (Cu), an additive in PE and PP microplastics. Their results showed a decrease in the intensity of the carboxyl groups and the loss of sulfone groups in *Spirulina* sp. introduced by Cu (II) at peaks from 830 to 862 cm^−1^. The loss of carboxyl and sulfone groups is the biggest factor in the decrease in phycocyanin in *Spirulina* sp., where these two groups are part of the phycocyanin structure, as shown in Figure 7. Besides the carboxyl and sulfone groups, the amine (NH_3_) and amide (CN, NH) groups associated with nitrogen also affected phycocyanin production.

The decrease in phycocyanin can be attributed to the availability of nitrogen within the biomass culture of *Spirulina* sp. after PE and PP microplastics are added. Sufficient availability of nitrogen may spur on the growth of *Spirulina* sp. and help produce good-quality biomass. However, a decreasing nitrogen concentration in the algae culture leads to disrupted photosynthesis by microalgae. This further leads to inhibited growth of *Spirulina* sp. and its biomass and subsequently decreased phycocyanin production. This chain of events is supported by Richardson et al. (1969), who studied the effect of nitrogen on the growth and composition of algae *Chlorella sorokiniana* and *Oocystis polymorpha*. They concluded that a nitrogen decrease of 4%–10% resulted in drastically reduced oxygen evolution, chlorophyll content, and production of microalgae tissue. The FTIR results on *Spirulina* sp. reflected this. They showed a decreased intensity of amine and amide groups after interacting with microplastics. The amine (NH_3_) and amide (CN, NH) groups in the biomass of *Spirulina* sp. are derivatives of nitrogen and important for the formation of phycocyanin compounds. The FTIR analysis of *Spirulina* sp. in (Figure 4) showed that the amine group (NH_3_) was detected at wavelength 3281 cm^−1^, the primary protein amide group (CN) at wavelength 1637 cm^−1^, and the secondary amide group (NH) at wavelength 1537 cm^−1^ (Dianratri et al., 2020). What followed was a change in the amine and amide organic compound groups, indicating the presence of nitrogen after treatment of *Spirulina* sp. with microplastics.

**Figure 8:**
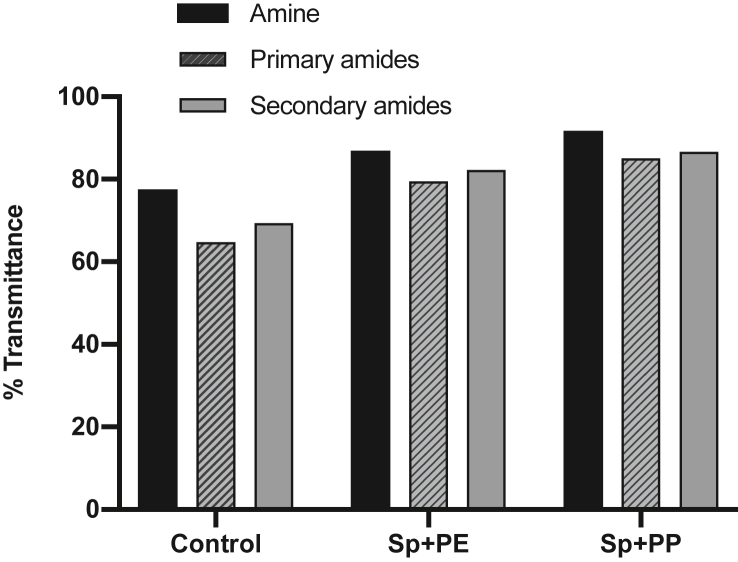
Changes in amine and amide groups showing the presence of nitrogen in *Spirulina* sp. before and after treatment with PP and PE microplastics.

The results of amine and amide groups in (Figure 8) showed that the %T of nitrogen increased after the addition of microplastics, as follows: %T of *Spirulina* sp. with PP treatment > %T of *Spirulina* sp. with PE treatment > %T of *Spirulina* sp. control. The increase in %T was inversely proportional to the decreased amine and amide group intensity linked to decreased nitrogen and phycocyanin formation. Thus, when %T increases, the nitrogen content decreases. According to Pavia et al. (2014), a %T nearing 100% is indicative of degradation caused by the decreased intensity of microalgal cell components. This was supported by previous research by Boussiba and Richmond (1980), who reported that phycocyanin, a protein compound in *Spirulina* sp., may degrade due to nitrogen deficiency. Furthermore, Safi et al. (2014) examined the effect of nitrogen contents on the total protein production from several types of microalgae. Their results showed that a decrease in nitrogen is directly proportional to the decrease in total proteins produced. Recently, Baroni et al. (2020) examined extracellular organic matter (EOM), specifically the protein and carbohydrates, in several microalgae with a ratio of nitrogen supply in kultur. The result is that nitrogen-deficient microalgal cells had less EOM (protein) per unit cell than when filled with nitrogen.

The results in Figure 8 prove that the presence of PP and PE microplastics can result in reduced growth and damaged *Spirulina* sp. cells and may negatively affect the nitrogen levels used by *Spirulina* sp. to form protein compounds, specifically phycocyanin. When microalgae are cultured in nitrogen-deficient conditions, the most prominent effect is a decrease in phycocyanin intensity, because pycocyanin is the most important pigment in microalgae *Spirulina* sp. and may reach 20% in dry weight of the cell protein (Hadiyanto et al., 2016).

## 4 Conclusion

The 30-day interaction between PE and PP microplastics and *Spirulina* sp., led to cracks in the surface of microplastics, and the emergence of new functional groups (carbonyl, carboxylic acid, hydroxyl, etc.) indicated that microplastics were degraded by *Spirulina* sp and microplastics also can significantly inhibit *Spirulina* sp growth (p < 0.05). A major factor in the decreased growth rate of *Spirulina* sp is the damage to *Spirulina* sp. cells due to microplastic additive particles that attack the cell surface during the microplastic degradation process and disrupted photosynthesis due to reduced light. The FTIR analysis showed that the decreased growth rate was directly proportional to the quality of Spirulina sp. biomass. All organic groups (especially proteins and polysaccharides) showed a decrease in intensity with PE treatment, and the intensity of organic groups was not detected with PP treatment. The decreased intensity of the organic functional groups greatly affected the quality of the biomass produced. The phycocyanin content in *Spirulina* sp. was similarly affected. The loss of carboxylate and sulfonyl groups showed that the presence of proteins in the biomass was the primary cause for the decreased phycocyanin content. *Spirulina* sp. and phycocyanin are often used in various industries, especially in the food industry. They must be free of contaminants, including microplastics that cause economic losses because it becomes unsafe and unfit for sale for consumption. Thus, further research is needed to identify the type of additive released by microplastic and its mechanism in inhibiting microalgae growth.

## Declarations

### Author contribution statement

H. Hadiyanto: Conceived and designed the experiments.

Inggar Dianratri: Performed the experiments.

Suherman Suherman, Fuad Muhammad & Adian Khoironi: Analyzed and interpreted the data.

Seetharaman Vaidyanathan: Wrote the paper.

### Funding statement

This work was supported by 10.13039/501100005844Universitas Diponegoro, Indonesia through World Research Research Undip (WCRU) under research grant number: 118-18/UN7.6.1/PP/2021.

### Data availability statement

Data will be made available on request.

### Declaration of interests statement

The authors declare no conflict of interest.

### Additional information

No additional information is available for this paper.
